# Forearm and Arm Tourniquet Tolerance

**DOI:** 10.5435/JAAOSGlobal-D-21-00229

**Published:** 2022-02-14

**Authors:** Rachel Lefebvre, Landon Cohen, Harrison Ford Kay, Amir Mostofi, Alidad Ghiassi, Milan Stevanovic

**Affiliations:** From the Department of Orthopaedic Surgery, Division of Hand Surgery, University of Southern California, Los Angeles, CA.

## Abstract

In distal upper extremity surgeries, there can be a choice to use an upper arm or forearm tourniquet. This study examines discomfort and tolerance in healthy volunteers to determine whether one is more comfortable.

**Methods:**

Forty healthy, study participants were randomized to an upper extremity laterality and site. Tourniquets were inflated to 100 mm Hg over systolic blood pressure. Participants experienced an upper arm and a forearm tourniquet sequentially. Visual analog scores (VAS) were recorded at 2-minute intervals. Time until request and VAS at tourniquet deflation were recorded. Time until the complete resolution of paresthesias was also recorded. Participants subjectively stated which tourniquet felt more comfortable.

**Results:**

Tourniquets were inflated longer on the forearm than the upper arm (mean 16.1 minutes versus 12.2 minutes; *P* < 0.0001). VAS at tourniquet removal was not different between the sites (means 7.3 and 7.3) (*P* = 0.839). Time until paresthesia resolution after the tourniquet was deflated was not different (means 8.1 and 7.7 minutes) (*P* = 0.675). Time until paresthesia resolution was proportional to tourniquet inflation time for both sites (regression coefficient 0.41; *P* < 0.00001). Participants found the forearm more comfortable (95% confidence interval, 0.63 to 0.92).

**Conclusion:**

Forearm placement allows the tourniquet to be inflated for an average of 4 minutes longer. Forearm tourniquet is subjectively more comfortable.

Tourniquet use has long allowed better visualization of structures allowing safe, precise, and efficient upper extremity surgeries.^1-4^ Many procedures can be done without a tourniquet using a wide awake, local anesthesia, no tourniquet (WALANT) technique. However, there are still some practice settings, patient characteristics, or procedural demands that necessitate the use of an upper extremity tourniquet.^5,6^ Particularly in more distal upper extremity procedures, there can be a choice about where to place the tourniquet: on the upper arm or on the forearm.

A review of the current literature indicates that not only site but also laterality, pressure, duration, and padding have been questioned. Previous studies in healthy volunteers have indicated that the upper extremity side, tourniquet width, and tourniquet inflation pressure do not influence the amount of discomfort or duration of tourniquet inflation.^7^ In multiple clinical studies, the anatomic site—forearm or arm—has not been found to influence the patient's blood pressure, heart rate, or pain scores while undergoing a carpal tunnel release under local anesthesia.^8-10^ Trials with small numbers of healthy volunteers, now done decades in the past, have disparate results on forearm versus arm tourniquet placement in relation to patient comfort.^10,11^ One report in the anesthesia literature provides evidence that Bier blocks are better tolerated with forearm rather than arm tourniquet placement.^12^

The goal of this study was to evaluate tourniquet discomfort, both by visual analog scores (VAS) recorded at regular intervals and by the amount of time healthy participants tolerated an inflated tourniquet, in both the forearm and upper arm using modern equipment and currently used tourniquet pressures. Uncomfortable numbness and paresthesias are present after tourniquet deflation, and so this study also sought to understand the length of this discomfort after the tourniquet has been released from different anatomic sites. The hypothesis is that there would be no difference in tourniquet tolerance measured using VAS and inflation time between the forearm and arm sites. We further hypothesized that paresthesias would resolve in the same time-dependent manner after the tourniquet release from either site.

## Methods

After Institutional Review Board approval, recruitment flyers were posted inviting study participation. Forty consecutive participants were enrolled. Patients saw the flyers when they came to the orthopaedic clinic for nonupper extremity issues. A standard script was used to describe the study after participants were screened. To be included, participants needed to have two well-perfused upper extremities and a normal basic screening neurovascular examination (<2 second capillary refill, 2+ radial artery pulse, and subjectively normal sensation throughout the hand and forearm). Participants had to provide written informed consent. Exclusion criteria included those with pre-existing pain, neurological symptoms, or a history of injury within the past 3 months in either upper extremity. Any participant with an abnormal attending-performed screening neurovascular examination would also be excluded and referred for additional appropriate care.

Informed written consent was obtained from all individuals participating in this study. Potential complication risk of the tourniquet use was discussed with all study participants.^3,13-15^ No tourniquet was inflated for longer than 60 minutes because this is the literature-accepted, low end of the safe upper time-range limit for an upper extremity tourniquet to be inflated.^3,16,17^ All tourniquet machines used were calibrated and in regular clinical use according to our operating room and University safety guidelines. All participants were observed after the tourniquet use until they objectively and subjectively returned to normal neurovascular baseline. Participants were contacted the following day, and all reported subjectively normal extremities.

To begin the study, participants' blood pressure was recorded. Participants were randomized with a random number generator to having the tourniquet first placed on their right or left upper extremity and to whether the tourniquet would be placed on the upper arm or the forearm.

Tourniquet inflation pressure was set to 100 mm Hg higher than the measured systolic blood pressure. This is the authors' standard practice and has been reported in the literature.^10,14^ The role of under tourniquet padding has been examined in the lower extremity literature with conflicting results.^18,19^ As is a standard practice at our institution, a 4-inch wide cotton Webril was wrapped around the planned tourniquet site so that a thickness of three Webril layers would be underneath the tourniquet and its edges. All participants had an 18-inch standard, commercially available tourniquet placed (Stryker). The 18-inch tourniquet appropriately fit all participants.

Study participants were seated in a chair with an adjustable height table positioned in front of them that was raised to the level of their heart. The VAS score before tourniquet inflation was recorded. Study personnel then held the patient's arm above the level of their heart for 60 seconds to allow for gravity exsanguination, and the tourniquet was inflated to the appropriate pressure. We chose not to exsanguinate with an Esmarch because we inherently could not control for variability in Esmarch pressure during application among anatomic sites and participants. The arm was then placed on the table to rest at the level of the heart during tourniquet inflation.

Participants were allowed to engage in light conversation with study personnel or look at personal reading materials. They were prompted to report their VAS every 2 minutes by study personnel and given the opportunity to review a sheet with the VAS scores and accompanying faces on it before reporting their score. The tourniquet was released at any point the participant requested. As part of the standardized script, patients understood that they could ask for the tourniquet to be deflated when they felt “uncomfortable”—a term purposefully left up to the participants' individual interpretation.

At the time the participant requested the tourniquet be let down, the tourniquet was immediately deflated and the total time of inflation was recorded. VAS just before deflation was recorded. The time until paresthesias and discomfort completely resolved after the tourniquet was deflated was also recorded. The second anatomic site was tested after all paresthesias from the first site completely resolved. No participant left the study site without reporting that their extremity felt “normal” to them. Participants were all contacted the day after the study to inquire about any residual discomfort or symptoms.

Participants were compensated $75 for their time if they participated in both upper and lower arm tourniquet placement, regardless of how long the tourniquets were left in place. All study participants completed the study, meaning that they participated in two tourniquet inflations: one upper arm and the other forearm. Although no participants chose this option, participants were offered $35 if they participated in only one tourniquet placement.

A paired Student *t*-test was used to compare the total time of tourniquet inflation on the upper arm and forearm of each subject and to investigate whether the order of inflation influenced the tourniquet time. A paired Student *t*-test was also used to investigate differences in the final pain scores reported at the time of tourniquet deflation from the forearm and the upper arm. Comparison of the time required for complete paresthesia resolution after tourniquet deflation between the two anatomic sites was also investigated using a paired Student *t*-test. A paired Student *t*-test was also used to determine whether the tourniquet order (side or anatomic site) was related to the time the tourniquet was inflated. A linear regression model was used to examine the relationship between the length of paresthesias after tourniquet deflation and the time the tourniquet was inflated.

## Results

Of 40 participants, 21 were female and 19 were male. The average age was 25.6 years (range: 23 to 33 years). The tourniquet pressure, on average, was 218 mm Hg (range, 197 to 238).

**Figure 1:**
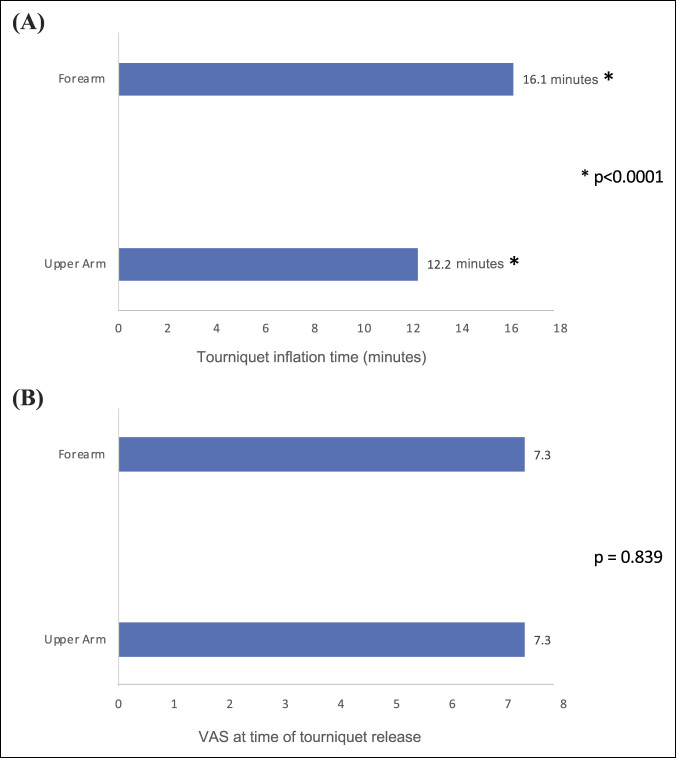
Bar diagram showing that tourniquet tolerance time varied significantly by the anatomic site with forearm tourniquets tolerated by participants for longer (mean 16.1 minutes, range 3.5 to 38.1 min, SD 9.2) than tourniquets on the upper arm (mean of 12.2 min, range: 3.1 to 32.9 min, SD 7.9) (*P* < 0.0001) (**A**). In both anatomic sites, similar VAS prompted participants to request tourniquet removal, despite different times of inflation (forearm mean 7.3, SD 1.36; upper mean 7.32, SD 1.19) (*P* = 0.839) (**B**). VAS = visual analog scores.

All participants completed forearm and upper arm tourniquet inflation. Tourniquets were inflated for a range of 3.1 to 38.1 minutes. Participants permitted the tourniquet to be inflated for a longer period on the forearm (mean 16.1 minutes, range: 3.5 to 38.1 min, SD 9.2) than the upper arm (mean of 12.2 min, range: 3.1 to 32.9 min, SD 7.9) (*P* < 0.0001) (Figure 1, A). Whether the upper arm or forearm tourniquet was inflated first or second did not significantly influence the duration the tourniquet was left inflated (first site mean = 13.7 minutes, SD 8.3; second site mean = 14.6 minutes, SD 9.2; t = 0.85; *P* = 0.399).

The VAS at the time the tourniquet was removed were not significantly different between the forearm (mean 7.3, SD 1.36) and upper arm (mean 7.32, SD 1.19) locations (*P* = 0.839) despite the difference in time that the tourniquet had been inflated (Figure 1, B).

**Figure 2:**
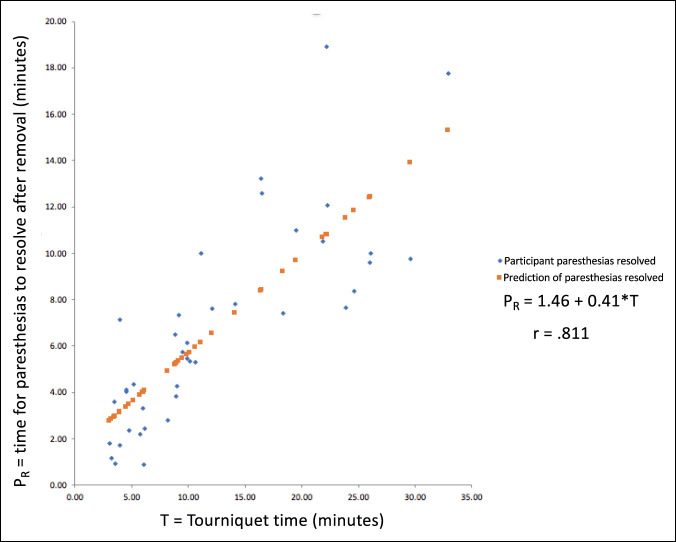
Graph showing that the time for paresthesias to completely resolve after tourniquet removal was not significantly different between forearm (mean 8.07; SD 5.07) and upper arm (mean 7.74, SD 4.47) locations (*P* = 0.675). For both anatomic sites, a regression coefficient of 0.41 is statistically significant (0 < 0.0001), indicating that it takes 0.41 minutes (or approximately 25 seconds) for paresthesia resolution after each minute of tourniquet inflation.

The time for complete paresthesia and discomfort resolution after the tourniquet was let down was not significantly different between the forearm (mean 8.07, SD 5.07) and upper arm (mean 7.74, SD 4.47) sites (*P* = 0.675). For both anatomic sites, a regression coefficient of 0.41 is statistically significant (0 < 0.0001) when investigating the time it takes for numbness to resolve after tourniquet removal (Figure 2).

When asked which tourniquet site was more comfortable, 31 of 40 participants chose the forearm site. The other nine participants felt the upper arm site was more comfortable. In statistical analysis, the forearm location was found to be markedly more likely to be comfortable for participants (95% CI 0.63 to 0.92).

## Discussion

An overarching goal during upper extremity surgery is to minimize patient discomfort both for patients who are awake and for those under sedation. When safe and possible, WALANT offers a tourniquet-free option for anesthesia and visualization during upper extremity surgeries. However, there is still a role for tourniquet use in awake and sedated patients with particular comorbidities, in certain practice settings, and in specific surgical procedures.^5,6^ Data from this study support the selection of the forearm tourniquet location over the upper arm location when possible.

This research was conducted in healthy volunteers not undergoing a simultaneous procedure. In previous clinical studies, forearm tourniquets have been described as “in the way” both for surgical access and draping and for finger position if the forearm tourniquet is contributing to pressure on the extrinsic finger flexors.^8,9^ Finger position and safe surgical exposure are critical considerations that may necessitate upper arm tourniquet placement in some cases. As our understanding of the overall patient perioperative experience evolves, a more distally draped out extremity with a surgical assistant or lead hand positioning the fingers appropriately may prove less disturbing for the patient-surgeon team overall.

Of particular note, the VAS score of 7.3 was constant across anatomic sites as the average uncomfortable level where participants requested tourniquet release. The average of 4 minutes longer than it took for the forearm tourniquet location to reach this VAS may make the clinical difference between requiring sedation to finish a procedure and being able to safely complete the surgery without additional anesthesia. Proponents of WALANT will note that in cases where a tourniquet can be safely avoided, patients can circumvent exposure to a VAS entering the “very severe” category.

In contrast to previous reports, our research found that the time for paresthesia resolution after tourniquet removal was dependent on the time of inflation. For each minute of tourniquet inflation, it takes about 25 seconds for paresthesias to resolve (Figure 2). This complements our understanding of the sensitivity of specific nerves to tourniquet ischemia.^11^ It can also guide expectations for paresthesia resolution after tourniquet release.

One potential weakness of our study is the unaccounted for psychological motivation of our participants and how this could differ from the motivation in patients undergoing a procedure. Our subjects knew that they would be compensated for their participation independent of tourniquet time. It is difficult to know if some participants wanted to maximize their “hourly rate” and request tourniquet release shortly after inflation or if participants aimed to please the study personnel and tried to extend tourniquet time as long as possible. Another weakness of this study is that it is not adequately powered for the continuous outcome of tourniquet time tolerance. An additional weakness of this study includes our young average age of subjects who consecutively presented to participate. The authors do not typically base their decision for using a tourniquet or not on age alone, and in clinical practice, a wider variety of ages would encounter tourniquet use in upper extremity surgery. Because of the young average age in this study, we cannot draw conclusions about variability in tourniquet tolerance and age.

Future studies may also consider the effect of the environment on tourniquet tolerance time, both in the operating room or in an experimental setting. Past studies in this arena do not describe whether participants were allowed to engage in distracting activities and how this may influence tourniquet tolerance.

## Conclusion

In conclusion, healthy volunteers request both arm and forearm tourniquet release as VAS approach the “very severe” pain with an average score of 7.3. It takes, on average, 4 minutes longer for forearm tourniquets to reach this level of discomfort when compared with upper arm tourniquets. Time for paresthesias to resolve after tourniquet removal is directly correlated with the inflation time, taking approximately 25 seconds for each minute of inflation for paresthesia resolution. Our study participants markedly preferred the forearm location rather than the upper arm location.

If a tourniquet needs to be used in upper extremity surgery, our data provide evidence that the forearm placement is better tolerated than the upper arm placement.
